# Going against the Grain: Atomistic Modeling of Grain Boundaries in Solid Electrolytes for Solid-State Batteries

**DOI:** 10.1021/acsmaterialsau.3c00064

**Published:** 2023-10-05

**Authors:** James A. Dawson

**Affiliations:** † Chemistry − School of Natural and Environmental Sciences, Newcastle University, Newcastle upon Tyne NE1 7RU, United Kingdom; ‡ Centre for Energy, Newcastle University, Newcastle upon Tyne NE1 7RU, United Kingdom; § The Faraday Institution, Didcot OX11 0RA, United Kingdom

**Keywords:** grain boundaries, solid electrolytes, density functional theory, molecular dynamics, ion transport, dendrites

## Abstract

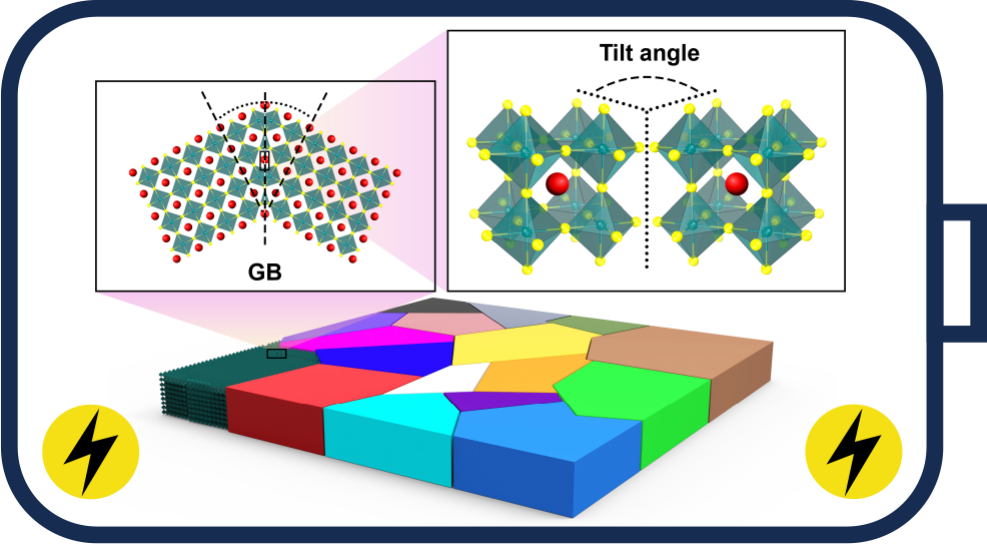

Atomistic modeling techniques, including density functional theory and molecular dynamics, play a critical role in the understanding, design, discovery, and optimization of bulk solid electrolyte materials for solid-state batteries. In contrast, despite the fact that the atomistic simulation of microstructural inhomogeneities, such as grain boundaries, can reveal essential information regarding the performance of solid electrolytes, such simulations have so far only been limited to a relatively small selection of materials. In this Perspective, the fundamental properties of grain boundaries in solid electrolytes that can be determined and manipulated through state-of-the-art atomistic modeling are illustrated through recent studies in the literature. The insights and examples presented here will inspire future computational studies of grain boundaries with the aim of overcoming their often detrimental impact on ion transport and dendrite growth inhibition in solid electrolytes.

## 1 Introduction

It is widely recognized that substantial improvements in energy and power densities, cost, safety, and lifetime are critical for our future energy demands. These performance requirements have driven the development of alternative battery technologies with potentially transformative performance that greatly exceeds current commercial rechargeable batteries.^1−5^ Of the many battery architectures currently being developed, one of the most promising is the solid-state battery,^6−10^ which utilizes solid electrolytes in contrast to the highly flammable liquid electrolytes used in conventional batteries. As a result, solid-state batteries are considered to be safer than their liquid electrolyte-based counterparts and also offer potential energy benefits from the use energy dense metallic anodes.^11−13^ Furthermore, the possibility of rapid charge and discharge resulting from the enhanced power density of solid-state batteries is another salient example of their promise. Nevertheless, despite these substantial benefits, solid-state batteries have several fundamental weaknesses that need to be overcome before their widespread utilization can be considered.^10,14,15^ Two of the most pressing of these are achieving sufficient ion transport across the whole device^6^ and the formation and propagation of metallic dendrites,^16^ which can lead to short-circuiting. Grain boundaries (GBs) are critical to both of these pertinent challenges.^17−19^

The solid electrolytes used in solid-state batteries are generally inorganic ceramic, polymer based or hybrid polymer–ceramic composites.^20^ Inorganic solid electrolytes, such as Li_7_La_3_Zr_2_O_12_, Li_10_GeP_2_S_12_, and Li_3_InCl_6_, are usually polycrystalline and contain high densities of GBs as a result of their synthesis from powders by sintering or pressing.^19^ GBs represent surfaces of contact between grains of different orientation and therefore have different local structures and compositions compared to the respective bulk materials.^6,21^ In the context of solid electrolytes, GBs can have vastly different ionic conductivities, electronic structures, and mechanical properties, thereby significantly influencing the macroscopic performance of not only the material but also the device. For example, it is well-known that the GBs in oxide solid electrolytes are typically highly resistant to ion transport, while their impact in sulfide solid electrolytes is often negligible.^6,17,18,22^ Furthermore, GBs are known to act as pathways for lithium and sodium dendrite growth, thereby potentially leading to short-circuiting and battery failure.^16,18,23,24^ GBs are therefore generally considered as detrimental to solid electrolyte and solid-state battery performance. Despite the overt importance of GBs for the design of high-performance solid-state batteries, their atomistic understanding remains limited compared to bulk materials due to the challenges associated with their experimental characterization and computational simulation.^18,19^ Hence, the development of novel methods to explore GBs and polycrystalline materials is pivotal.

**Figure 1:**
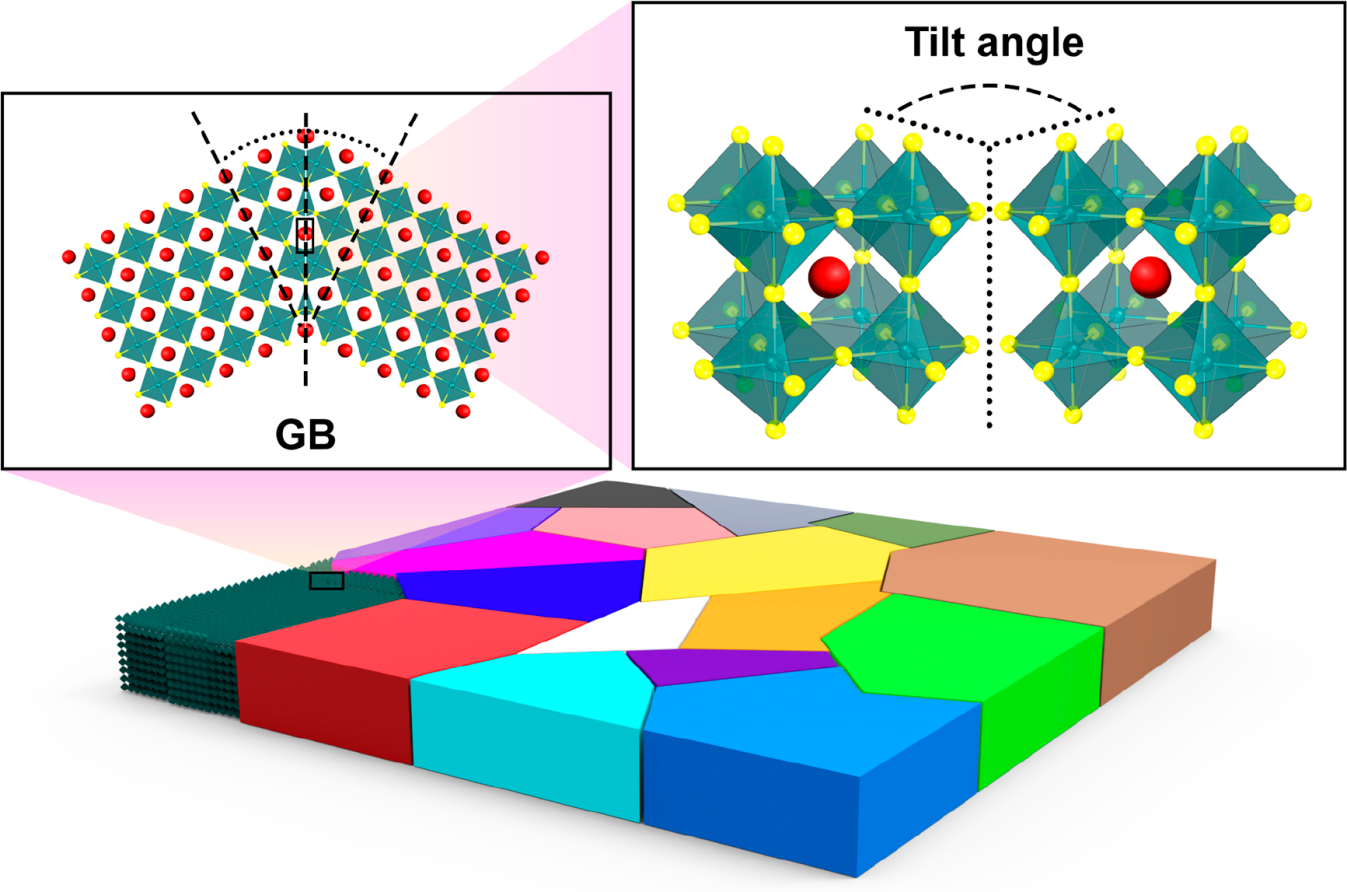
Schematic illustration of the atomistic modeling of individual GBs and polycrystals.

The application of atomistic modeling has been central to the progress made so far for solid electrolytes and solid-state batteries. Density functional theory (DFT), ab initio molecular dynamics (AIMD), and force field-based molecular dynamics (MD) simulations have all been extensively used to explore ion transport mechanisms,^25−27^ stability,^28−30^ electronic structure,^31−33^ defects,^27,34,35^ and doping^36−38^ in bulk solid electrolytes and at their interfaces with electrodes. In contrast, the use of these techniques to explore such properties in the GBs of solid electrolytes for solid-state batteries is only now becoming mainstream, with all studies coming in the last six to seven years. Atomistic simulations have so far been used to investigate two main areas of importance for solid-state batteries, namely, ion transport and dendrite formation and propagation, in both representative GB and complete three-dimensional polycrystalline structures (see Figure 1). The vast array of properties that can be calculated through such GB simulations include ionic diffusion coefficients (and conductivities), activation energies, GB stabilities, electronic structure and transport, and mechanical properties. The modeling of GBs can therefore provide a wealth of important information, some of which is not currently accessible experimentally, at atomic resolution.

In this Perspective, the fundamental understanding and progress that have been achieved so far for solid electrolytes and solid-state batteries through the atomistic simulation of GBs are highlighted with recent examples from the literature. The major computational techniques used to investigate GBs and polycrystals at the atomistic scale and the information that they can reveal are first introduced. Given the role that GBs can have in determining the overall ionic conductivity of a solid electrolyte, it is unsurprising that most computational studies focus on this key topic, as presented and discussed here with key examples. Next, the application of modeling in determining the mechanical and electronic properties of GBs and how they contribute to dendrite growth in solid-state batteries are considered. Finally, potential future trends, opportunities, and challenges for the atomistic simulation of GBs are proposed in the context of developing state-of-the-art solid electrolytes and solid-state batteries.

## 2 Computational Methods

Both first-principles (e.g., DFT and AIMD) and force field-based (e.g., MD) methods have been widely utilized in the study of GBs in solid electrolytes. Only a brief overview of the application of the atomistic simulation methods for GBs and polycrystals is provided here because more detailed reviews of the theory behind these techniques are widely available in the literature.^39,40^ The methods used for the construction of atomistic models of GBs and polycrystals are also presented.

### 2.1 First-Principles Methods

First-principles methods are based on the laws of quantum mechanics and describe the behavior of electrons, which in turn can be used to predict the structures and properties of materials. Of the various methods available, DFT is the most well-known and utilized for the simulation of inorganic solids, including solid electrolytes. The Schrödinger equation for a many-body system may be simplified to the Kohn–Sham equation (a single-particle-independent Schrödinger equation) and can be numerically solved with density functional theory. DFT has become ubiquitous in the calculation of electronic structure and now plays a pivotal role in the understanding, discovery, design, and optimization of new materials for a wide range of energy and chemical applications, including rechargeable batteries.^41^ For GBs, DFT simulations have been used to calculate, for example, the barriers for Li- and Na-ion hopping via the nudged elastic band method, density of states, bandgaps, polaron formation and transport, defect formation energies, stability, and mechanical performance.

AIMD simulations, where the classical Newtonian equations of motion for a system are solved numerically and the interatomic interactions are defined by first-principles calculations, have proven to be powerful for investigating long-range Li- and Na-ion transport at the GBs of solid electrolytes. Although computationally expensive and typically limited to hundreds of ions and time scales of hundreds of picoseconds, AIMD simulations can provide highly accurate potential energy surfaces of materials and be used to quantify diffusion coefficients directly as a function of temperature, which can then be plotted using the Arrhenius equation to obtain activation energies for ionic conductivity.^42^ AIMD calculations can be used to identify and understand the impact of GBs on ion transport compared to bulk materials. Specific examples of the use of DFT and AIMD simulations for solid electrolyte GBs are presented and discussed below.

It is anticipated that the role of first-principles simulations as mainstream tools for investigating GBs and other nano/microstructural inhomogeneities will only continue in forthcoming years, with the design of new functionals and methods with improved accuracy and reduced computational expense representing a major ongoing area of research interest.^43^ Of the many currently available DFT-based codes, the Vienna Ab Initio Simulation Package (VASP)^44,45^ is the most popular for the simulation of solid-state battery materials and their GBs.

### 2.2 Force Field-Based Methods

Even with the rapid increases in computational power and the exploitation of first-principles calculations, force field methods continue to play a major role in atomistic modeling. The reliability and accuracy of such methods are highly dependent on the force fields utilized. A classical force field describes the total energy of a system as a function of the nuclear coordinates. For ionic and semi-ionic solids, the total energy can be partitioned into two terms: a long-range Coulombic term and a short-range term that accounts for Pauli repulsion and covalent and dispersive attractive interactions. A range of force field forms are used to represent these short-range interactions, and they can be fitted to experimental data and/or results from first-principles simulations.^46−48^ Proven force fields are available for a wide range of solid electrolyte materials, including oxides^49,50^ and sulfides.^51,52^

The use of force field-based methods in materials modeling has experienced a renaissance in recent years because of the development of machine-learned force fields (MLFFs). MLFFs combine the accuracy of DFT simulations and the efficiency of classical force fields by learning the relationship between the chemical structure and potential energy of a system. The construction of MLFFs requires suitable reference data to learn the relevant structure–property relations, including energy, forces, or a combination of both, which are typically obtained from first-principles calculations. As a result, all of the chemical behavior is learned from the reference data. Powerful and accurate MLFFs have been developed for a range of topical solid electrolyte materials.^53−55^ Several exhaustive reviews of MLFFs and their development have been published in recent years.^56−58^

In the context of solid electrolytes and other battery materials, MD simulations are the most well-utilized application of such force fields. In an MD simulation, Newton’s equations of motion are solved using a numerical, iterative procedure over many time steps, leading to a clear image of the evolution of ion positions and velocities updated using the known velocities and forces, respectively, as a function of time. The chosen time step for an MD simulation must be shorter than the typical time associated with important processes, such as an atomic vibration, with values of 1 or 2 fs often utilized for solid electrolytes. MD simulations can provide an enormous amount of important data for analysis. In the context of GBs, MD simulations are most often used to produce diffusion coefficients for mobile ions and to analyze the ion transport mechanisms as a function of temperature. MD can also provide information on structural properties, for example, radial distribution functions and stability. The extensive repertoire of computational techniques implemented in the Large-scale Atomic/Molecular Massively Parallel Simulator (LAMMPS)^59^ for the treatment of materials and their dynamics make it a powerful tool for the modeling of solid electrolytes and ion conductors.

Force field-based MD simulations can easily reach the nanosecond range with simulation cells in excess of one million atoms when implemented on high-performance computing platforms, whereas AIMD simulations are limited in comparison with time scales in the picosecond range and simulation boxes of less than 1000 atoms. Nevertheless, AIMD calculations have the distinct advantages of explicitly including electronic structure and not requiring complicated force field fitting. Regardless of their individual strengths and weaknesses, both MD and AIMD approaches are widely used for the simulation of solid electrolyte materials and their interfaces.

### 2.3 Atomistic Modeling of Grain Boundaries and Polycrystals

Both first-principles and force field-based methods, as described above, can be used to model GBs at the atomistic scale. However, compared to the modeling of bulk crystalline materials, the modeling of GBs can be far more computationally expensive and requires several additional factors to be considered. While bulk crystalline materials are modeled as infinite lattices using three-dimensional periodic boundary conditions, GBs are typically two-dimensional and require the preparation of a surface slab that is placed in direct contact with another slab of the same material (see Figure 1). GBs are often categorized by the Miller indices (*hkl*) of the associated grains and a rotation angle, which indicates how much of the grain is rotated around the rotation axis. For example, symmetric tilt (twin) GBs, are formed by two grains with equivalent Miller indices and a rotation angle of 0°, while twist boundaries are characterized by a rotation angle with the rotation axis perpendicular to the boundary. Another important parameter used to describe GBs is Σ, which is effectively the reciprocal of the fraction of points that are coincident between differently oriented grains and represents a convenient indicator of whether a GB is high symmetry (low Σ) or low symmetry (high Σ). Perfect bulk materials are therefore considered to have a Σ value of 1. More details on the types, notation, and characterization of GBs are available elsewhere.^21^ Although the development of such interfacial models is challenging and careful consideration must be given to their scale, composition, and stability to ensure that they are reliable and representative of real GBs, they can offer a wealth of valuable information regarding the performance of materials and their unique behavior and properties.

**Figure 2:**
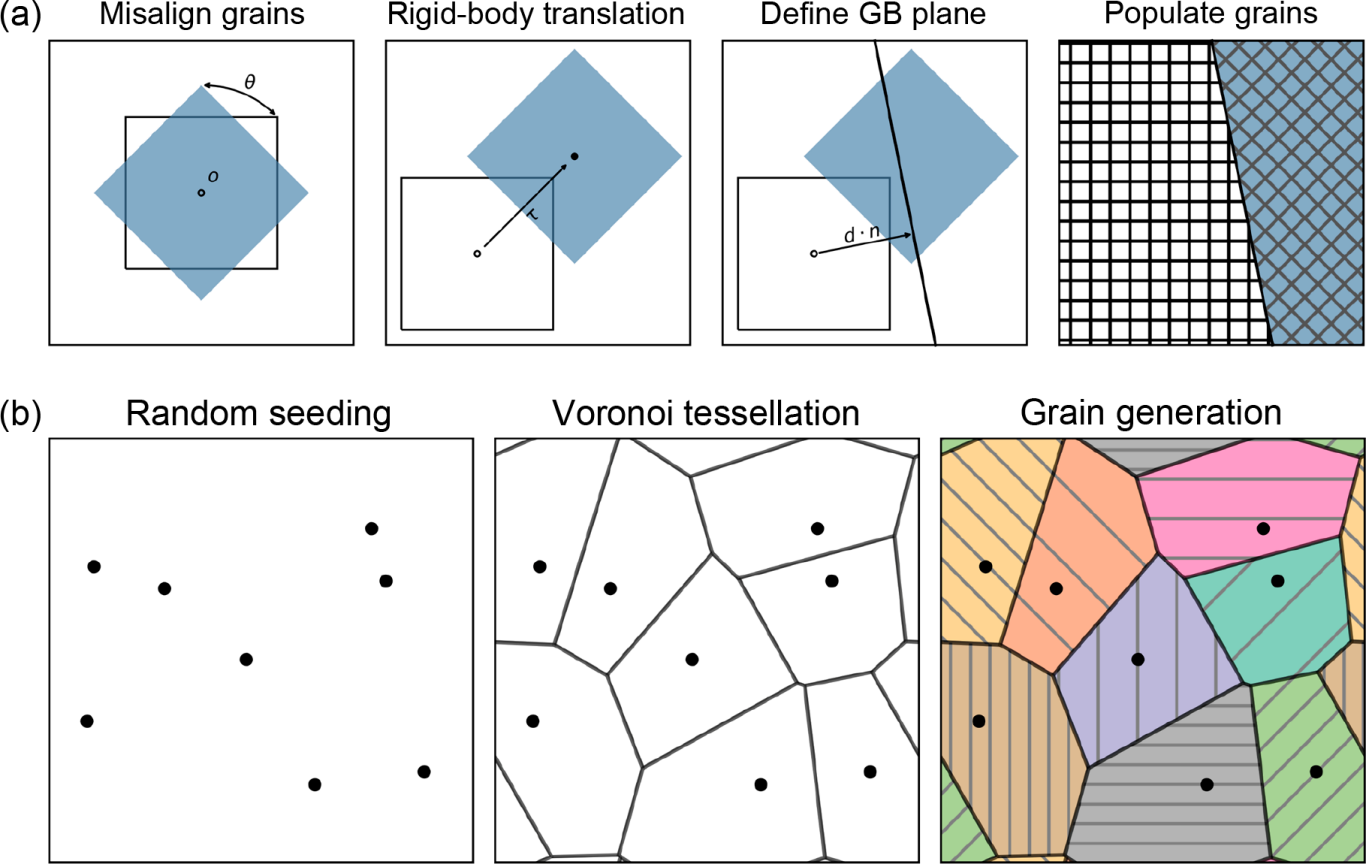
(a) Formation of a GB model where two lattices are misaligned by a tilt angle θ about a rotation axis *o*. An optional rigid-body translation τ of one grain relative to the other yields asymmetric GBs. The GB plane is defined by a normal vector *n* and distance scalar *d*. Atoms of each crystal are rejected based on their position relative to the GB plane. (b) Procedure for generating polycrystals where crystal “seeds” are distributed in a simulation box and randomly misoriented. Regions associated with each seed are determined using Voronoi tessellation to yield grain volumes. Each seed is expanded to populate each grain volume with atoms to yield a polycrystal.

As schematically presented in Figure 1, the creation of atomistic GB models has so far been carried out for both individual GBs and polycrystals containing a given number of randomly orientated grains and GBs, with the former being much more prevalent for solid electrolytes in the literature. The process for creating the GB models is outlined in Figure 2(a). Most individual GBs simulated for solid electrolytes are twin boundaries and are constructed using the coincidence site lattice theory,^21^ where two individual grains are tilted by a given tilt angle until their surface planes coincide. These structures are then modeled in a periodic supercell containing two grains of finite width brought into contact to produce two GBs. It is essential to ensure that the grains in each model are sufficiently thick to minimize the interactions between them. A systematic scan over all possible rigid-body translations between the grains is typically undertaken to identify low-energy GB structures. The structure with the lowest energy is determined to be a stable structure, where the formation energy, γ, in a periodic supercell is given by
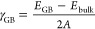
1where *E*_GB_ is the total energy of the GB system, *E*_bulk_ is the total energy of the bulk system, and *A* is the cross-sectional area of the slab, where the factor of 2 accounts for the two equivalent interfaces in the model. This procedure has been used extensively in both first-principles and force field-based studies to successfully predict and model GB structures.

Figure 2(b) depicts the method used to procedurally generate atomistic models of polycrystalline systems. Multiple seeds are distributed in a simulation cell with the position and orientation of these seeds randomized. The regions of space associated with each seed are then determined by Voronoi tessellation, yielding a grain for each seed. The system is then populated with atoms from the seed locations outward, emulating the growth of crystals in real systems. Given that it is not possible to ensure that low energy environments are generated by this approach (or indeed that the resultant polycrystal will be charge neutral), a large number of polycrystals may need to be generated and relaxed in order to find energetically feasible systems. Such polycrystals can be readily constructed using Atomsk,^60^ as a powerful set of tools for manipulating crystal structure files. One of the main advantages of this method compared to the modeling of individual GBs, as described above, is that it accounts for hundreds or thousands of GBs simultaneously and therefore enables the analysis of properties, including ion diffusion, as a function of grain size. However, the considerable size of such systems means that the use of first-principles simulations to investigate such systems is not viable, and the analysis of the MD output can be more challenging.

## 3 Modeling Ion Transport at Grain Boundaries

Given the significant impact that they can have on ion transport in solid electrolytes for solid-state batteries, it is logical that most atomistic modeling studies of GBs are focused on determining the rates and mechanisms of ion diffusion at these important interfaces. In this section, we highlight the progress that has been made so far regarding the computational understanding and manipulation of ion transport at and near GBs in solid electrolytes.

Two of the earliest studies to build atomistic models of GBs for battery solid electrolytes were carried out on important oxide systems, namely, garnet Li_7_La_3_Zr_2_O_12_ (LLZO)^61^ and antiperovskite Li_3_OCl.^17^ Both studies utilized classical MD simulations to investigate Li-ion transport in a range of large-scale GB models. In the work of Yu and Siegel,^61^ the Li-ion transport properties of three Σ3 and Σ5 model GBs in LLZO were studied, and it was found that Li-ion transport was generally slower in the GB region compared to the bulk depending on the GB structure and temperature. In our study,^17^ Li-ion conductivity was again found to be severely hindered through the Σ3 and Σ5 GBs of Li_3_OCl. The activation energies for Li-ion conduction across the GBs were consistently higher than those of the bulk system, thereby confirming the high GB resistance in this material. Based on our MD results, we also proposed a polycrystalline model to quantify the impact of GBs on conductivity as a function of grain size. Both of these studies have been critical in driving this field forward and have inspired numerous subsequent studies of a similar nature.

Since our initial work in 2018,^17^ several other computational studies have considered the influence of GBs on Li- and Na-ion transport in antiperovskite solid electrolytes. For example, Chen et al.^62^ used static DFT and AIMD simulations to again investigate the Σ3 and Σ5 GBs of Li_3_OCl. In agreement with our earlier study, it was found that these GBs present significantly lower Li-ion diffusion coefficients and higher activation energies compared to those of the bulk material. In a later study,^63^ we assessed the impact of the Σ3(111) GB on Li-ion transport in the hydrated antiperovskite Li_2_OHBr using DFT and AIMD simulations. The Li-ion diffusion coefficient of the GB at 300 K was almost 2 orders of magnitude lower than for the bulk material. A structural analysis of the GB revealed that its increased disorder prevented intergranular Li-ion diffusion, thereby effectively limiting Li ions to two-dimensional diffusion pathways. Van Duong et al.^64^ constructed explicit polycrystalline models for a range of Li- and Na-based antiperovskite solid electrolytes with the general formula A_3_OX (A = Li or Na; X = Cl or Br). From large-scale molecular MD simulations, their polycrystalline systems had higher activation energies for ion conductivity compared with values reported in previous theoretical studies that do not account for GBs. Unfortunately, no direct comparison to bulk materials was given.

**Figure 3:**
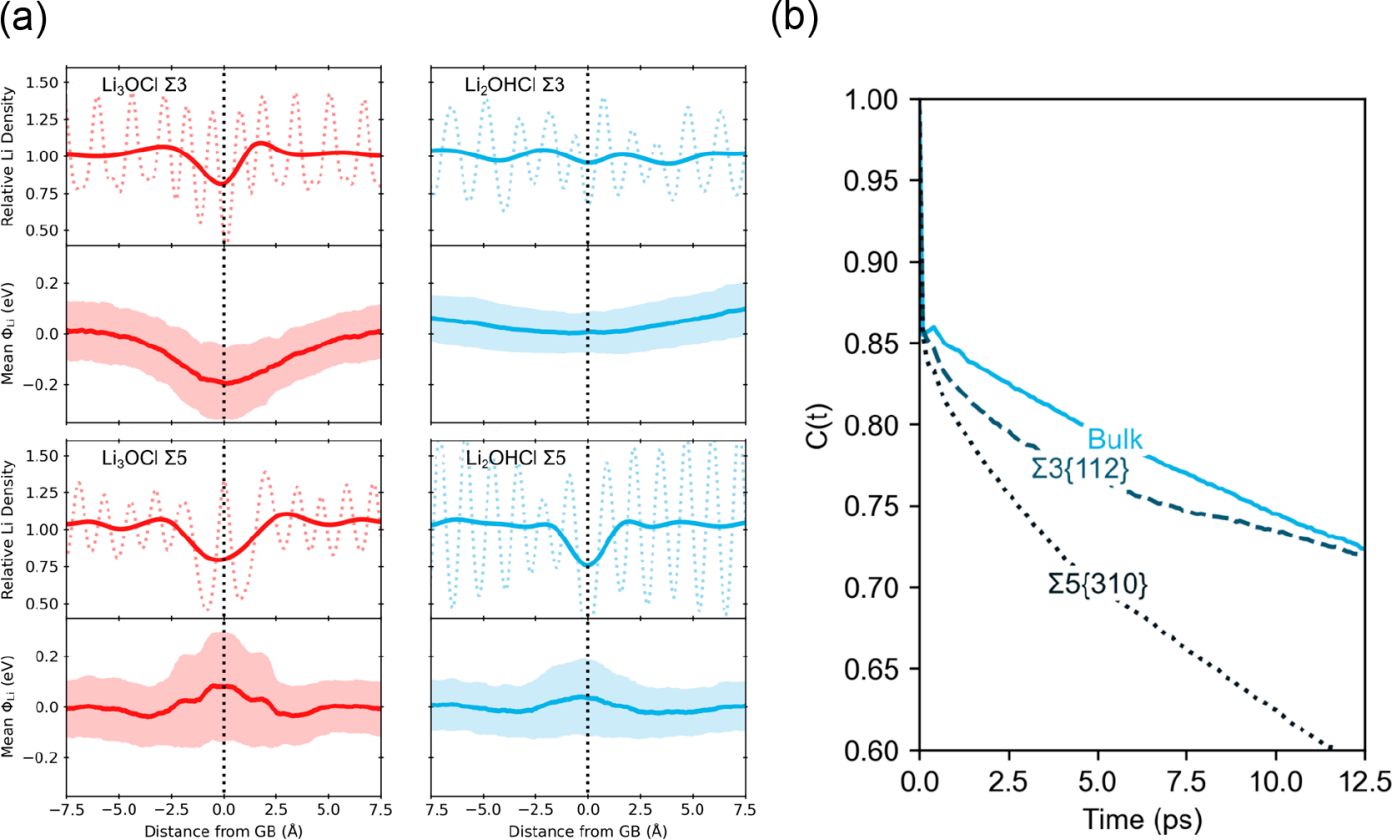
(a) Calculated relative densities of Li (top panels) and mean electrostatic potentials around Li ions, ϕ_Li_, (bottom panels) as a function of distance from the GB for the Li_3_OCl Σ3(112), Li_3_OCl Σ5(310), Li_2_OHCl Σ3(112), and Li_2_OHCl Σ5(310) GBs at 600 K. (b) Vector autocorrelation function, *C*(*t*), for OH^–^ rotation at the bulk and GBs of Li_2_OHCl. Reproduced with permission under a CC BY 4.0 license from ref (18). Copyright 2023, Wiley-VCH.

Finally, in a very recent study,^18^ we performed DFT and AIMD calculations to develop the first generally applicable design principles for GBs in solid electrolytes (Figure 3). Of the materials considered, it was found that the Li_3_OCl GBs pose large barriers to ionic conductivity whereas the GBs in Li_2_OHCl are comparatively benign, as illustrated by the difference in the electrostatic profiles around the Li ions at the GBs in these materials (Figure 3(a)). This difference was attributed to the OH^–^ anion with a strong dipole that can undergo rotation and reorientation to oppose the electric fields created by electrostatic perturbations in the vicinity of the GBs. This can effectively reduce the drive for Li vacancies to segregate and reduce the long-range impact that local structural changes can have on the electrostatic potential. Such anionic rotation is often referred to as the “paddlewheel effect” and has been widely studied in antiperovskites^65−67^ and other solid electrolytes.^68−70^ In this study, we investigated the role of the paddlewheel effect at GBs for the first time and observed much faster reorientation in the Σ5(310) GB of Li_2_OHCl (indicated by the rapid decrease in its vector autocorrelation function, *C*(*t*), in Figure 3(b)) compared to the bulk materials, representing another factor that can reduce interfacial resistance from GBs in Li_2_OHCl compared to Li_3_OCl.

**Figure 4:**
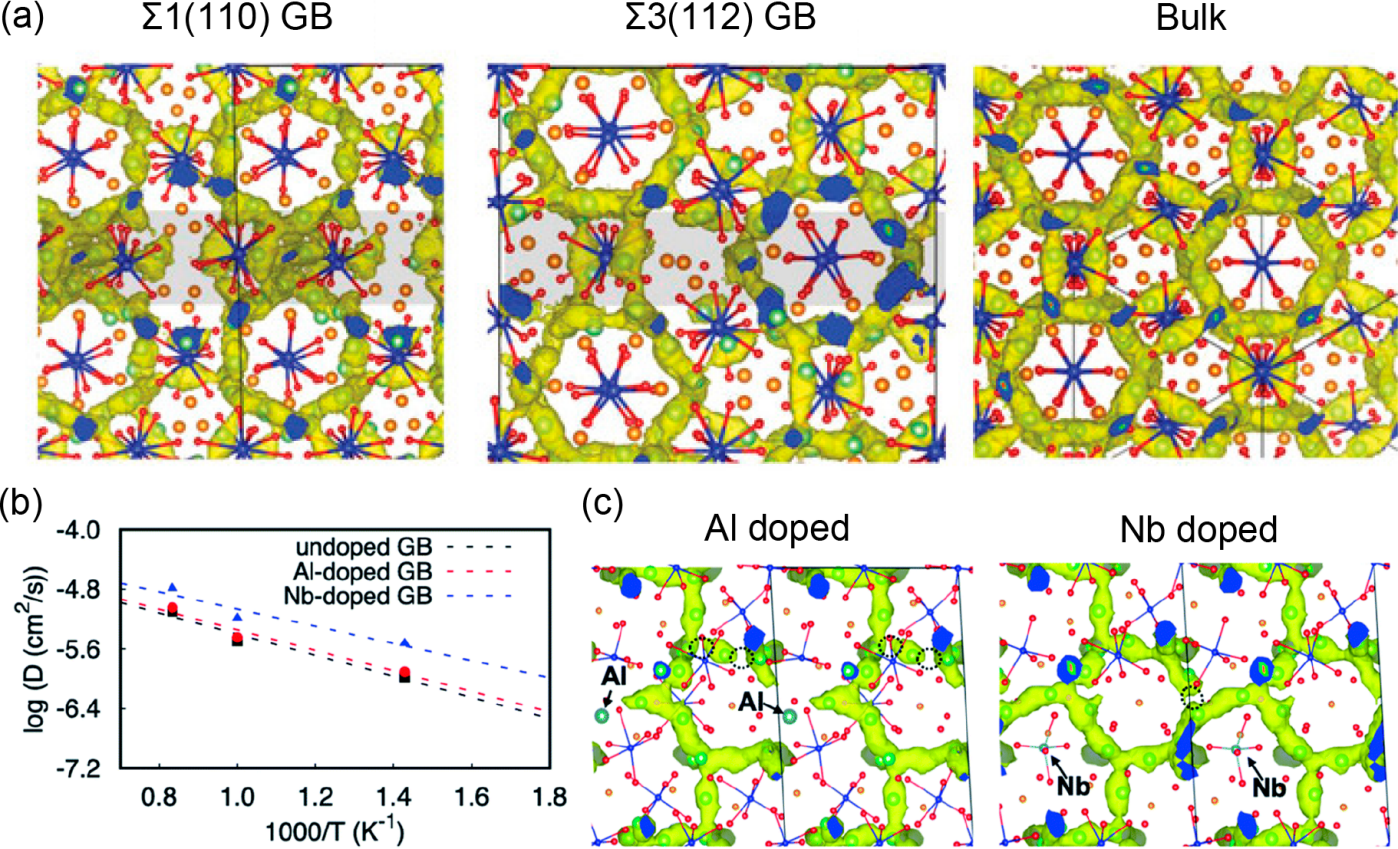
(a) Li-ion trajectory densities accumulated from AIMD simulations at 1000 K in Σ1(110) and Σ3(112) LLZO GBs and bulk LLZO. Reproduced with permission from ref (72). Copyright 2022, Wiley-VCH. (b) Arrhenius plots of Li-ion diffusion coefficients in undoped and Al- and Nb-doped Σ3(112) GB models of LLZO. (c) Partial Li-ion trajectory densities accumulated from AIMD simulations at 1000 K in Al- and Nb-doped Σ3(112) GB models of LLZO. The dashed circles indicate disconnection of the trajectory density. Reproduced with permission from ref (73). Copyright 2022, Royal Chemical Society.

Building on the early work of Yu and Siegel,^61^ one of the most studied solid electrolytes in terms of the simulation of GB properties has been LLZO. In the work of Shiiba et al.,^71^ MD simulations were carried out on a large range of cubic LLZO GBs with different symmetries. The results of the simulations revealed that although the Li-ion conductivity within and across the GB layer was generally reduced compared to the bulk conductivity, this effect was highly dependent on the off-stoichiometric Li-ion composition within different GB structures. Subsequently, Gao and co-workers^72,73^ used first-principles calculations to investigate Li-ion transport around GBs in LLZO. They reported that resistance in their LLZO GB models was highly dependent on their structures. In particular, their Σ3(112) GB showed similar conductivity to the bulk due to its bulk-like Li-ion migration network (see Figure 4(a)), whereas the Σ1(110) GB with distinct diffusion paths displayed a significantly lower Li-ion conductivity.^72^ The possibility of oxygen diffusion at an elevated temperature was also proposed. As displayed in Figure 4(b), the same authors again used DFT calculations to probe Li-ion transport at the GBs of LLZO doped with Al and Nb doped at the Li and Zr sites, respectively.^73^ AIMD simulations indicated that the segregation of Nb dopants at the GBs results in an improved Li-ion conductivity. Alternatively, the doping of Al at LLZO GBs was found to have a minimal impact on the Li-ion conductivity as a result of the immobile Al ions blocking nearby Li-ion hopping. DFT and AIMD simulations were also conducted for Ta-doped LLZO and it was again found that the GBs in this material result in significant interfacial resistance, possibly due to the formation of secondary phases with high Li-ion migration barriers.^74^

Perovskite oxide solid electrolytes have also received attention due to their GB properties. We carried out an atomistic modeling study of GBs in Li_3*x*_La_(2/3)–*x*_TiO_3_ (0 < *x* < 0.16, LLTO) using classical MD simulations.^75^ Our calculations revealed that the five studied GBs (Σ2(110), Σ3(111), Σ5(210), Σ3(211) and Σ5(310)) presented Li-ion conductivities that were 1 to 2 orders of magnitude lower than for the bulk. An increase in the Li-ion migration activation energy was observed for all grain boundaries compared to the bulk. This helped to rationalize why previously calculated activation energies for bulk LLTO have been consistently underestimated compared to experiment, similar to the above studies for other oxide solid electrolytes. In a more recent study, Lee et al.^54^ studied the impact of GBs on Li-ion transport in another oxide perovskite, Li_0.375_Sr_0.4375_Ta_0.75_Zr_0.25_O_3_ (LSTZ0.75), which, unlike LLTO, exhibits low GB resistance. Specifically, the authors used hybrid Monte Carlo/MD simulations enabled by an MLFF to investigate the structures and compositions of GBs in LSTZ0.75 to understand why they do not significantly reduce Li-ion transport, as is typical oxide solid electrolytes. It was found that the GBs in LSTZ0.75 maintained the perovskite framework, thereby preventing them from forming structures that generate new barriers for Li-ion transport. Furthermore, the additional Sr vacancies present at the GBs naturally increase the number of percolation pathways and facilitate Li-ion transport. These findings helped to explain the fundamental difference between the impact of grain boundaries on ionic conductivity in LLTO and LSTZ0.75.

A final category of oxide solid electrolytes that has been investigated computationally with regard to its GBs is the Na superionic conductor (NASICON) type. Nakano and coauthors performed two studies on the GBs of NASICON-type LiZr_2_ (PO_4_)_3_ (LZP) solid electrolytes.^76,77^ In the first study,^76^ the Li-ion conductivities of 32 GB structures were determined using a MLFF for LZP to obtain a comprehensive understanding of the effect of GB structures on Li-ion conductivity in this material. The calculated Li-ion conductivities of the GBs were in the wide range from 10^–7^ to 10^–4^ S cm^–1^ at room temperature. Given that the Li-ion conductivity of the bulk model was ∼10^–5^ S cm^–1^, it was predicted that some of the GB structures could actually be beneficial for Li-ion transport in LZP. The correlation between Li-ion conductivity and the cavity size around Li sites at the GBs was also confirmed, with a cavity size of 2.8 Å found to be optimal for Li-ion diffusion. In the subsequent study,^77^ the authors extended their work on LZP to polycrystalline models by developing a method to analyze the local ion flux from nonequilibrium MD simulations. The analysis of their results revealed several interesting findings; for example, the GB resistivity of their LZP polycrystals was estimated to be ∼30 times higher than that of the bulk, and Li ions were found to be strongly trapped at the sites in the vicinity of broken PO_4_ and/or ZrO_6_ network connections.

In contrast to oxide solid electrolytes, sulfide solid electrolytes generally present negligible GB resistance.^22^ This factor has resulted in far fewer atomistic modeling studies of GBs in sulfide compared to oxide solid electrolytes. Furthermore, most sulfide solid electrolytes contain polyanions, such as PS_4_^3–^, which can make it more challenging to construct stable GB models without breaking P–S bonds.^18^ Nevertheless, we have performed two studies in this area in recent years. Inspired by earlier experimental work showing that reducing the particle size Li_3_PS_4_ can result in orders of magnitude higher Li-ion conductivity,^78^ our first study^52^ was conducted to ascertain the effect of nanostructuring on Li-ion transport in Li_10_GeP_2_S_12_. This was achieved by using state-of-the-art MD simulations on nanocrystalline systems. Our results predicted that the Li-ion conductivity of Li_10_GeP_2_S_12_ increases with decreasing grain volume due to a fundamental change from a primarily one-dimensional Li-ion conduction mechanism to a three-dimensional mechanism and major changes in the local structure. The room-temperature Li-ion conductivity for the smallest nanometric particle size was three times higher than that of the bulk material. Our second study^18^ featured the archetypal Li-ion conducting sulfide solid electrolyte, Li_3_PS_4_, and used DFT and AIMD calculations to analyze the role of GBs in determining its short- and long-range Li-ion transport. In agreement with experimental findings for sulfide solid electrolytes, our calculations revealed only a minor impact from GBs on the Li-ion conductivity of Li_3_PS_4_, as reflected by the similar activation energies obtained for its GBs and bulk and the remarkably flat electrostatic profiles across its GBs. More interestingly, although we observed significantly more libration of the PS_4_^3–^ groups at the GBs compared with the bulk, it was found that this did not result in an increase in conductivity. Nevertheless, this finding may have important implications for other sulfides, particularly those with regions of low crystallinity, e.g., amorphous or glass-ceramic materials.

To the best of our knowledge, our recent work features the only atomistic study of GBs in a halide solid electrolyte, namely, Li_3_InCl_6_.^18^ Unlike for oxide and sulfide solid electrolytes, the influence of GBs on the ion transport in halide solid electrolytes is relatively unknown. In our DFT and AIMD study, we found that the interfacial diffusion behavior of Li_3_InCl_6_ shows an encouraging tolerance toward GBs, similar to that of sulfides. As for Li_3_PS_4_, Li_3_InCl_6_ showed a flat electrostatic profile, confirming that GBs do not have a strong impact on the electrostatic landscape of Li ions in this material. The variation in the density of Li ions across the grain boundaries of Li_3_InCl_6_ was also minor. We also investigated how GBs affect correlated Li-ion transport in Li_3_InCl_6_, and it emerged that their presence has no significant impact, with similar correlation time scales calculated for both the GBs and bulk.

**Figure 5:**
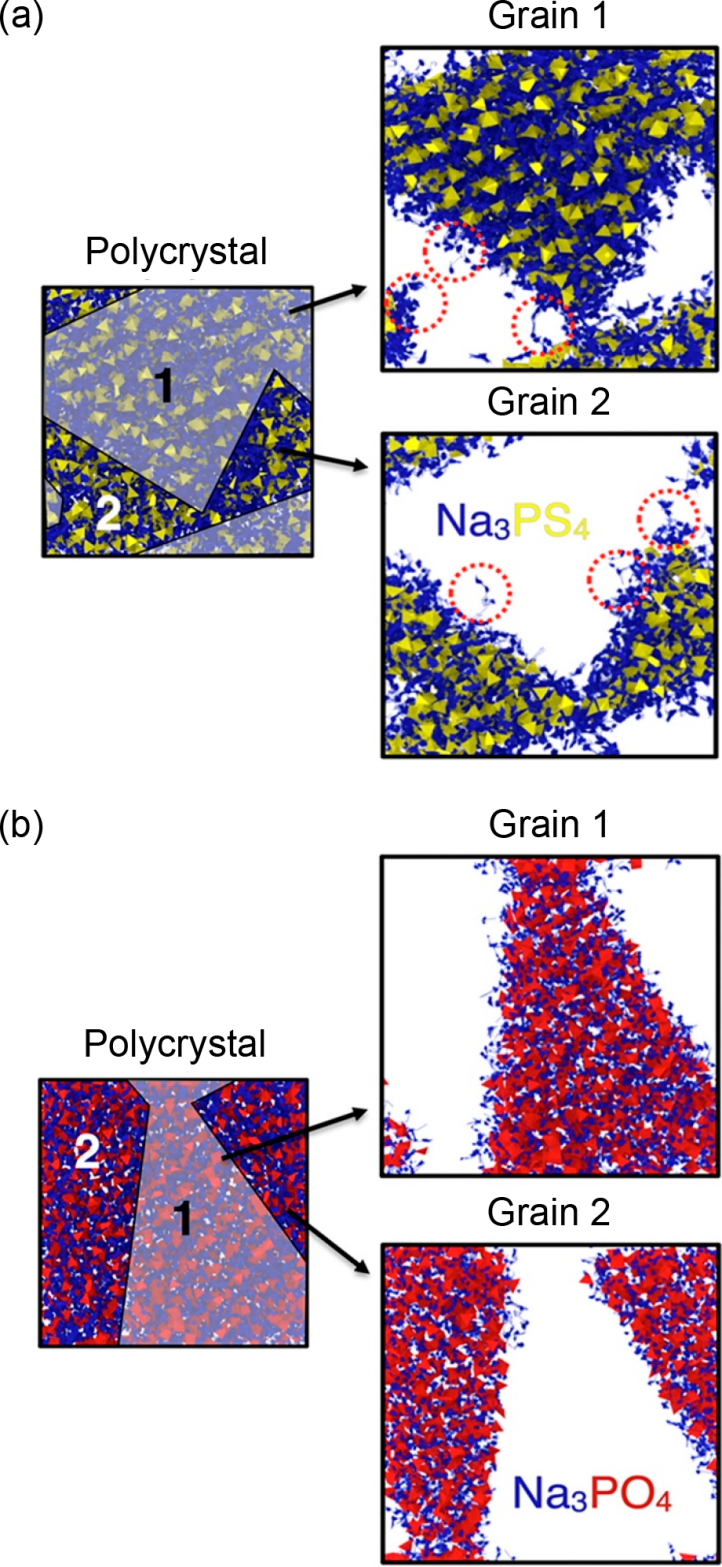
Diffusion density plots of Na ions (blue) overlaid on PS_4_ (yellow) and PO_4_ (red) tetrahedra in (a) Na_3_PS_4_ and (b) Na_3_PO_4_ polycrystals, respectively, with two grains at 400 K. Red circles highlight areas of significant intergranular diffusion. Reproduced with permission from ref (22). Copyright 2019, American Chemical Society.

While the number of studies featuring the computational characterization of GBs in Li-based solid electrolytes has increased rapidly in recent years, there are, to the best of our knowledge, only three equivalent studies of ion transport at the GBs of Na-based solid electrolytes. All of these studies focus on Na_3_PS_4_ as the archetypal Na-ion conducting sulfide. The first was carried by our team in 2019 and was also the first to develop explicit polycrystalline models to understand the different role of GBs in oxide- and sulfide-based battery solid electrolytes.^22^ As displayed in Figure 5, we used two model polycrystalline electrolyte systems, Na_3_PS_4_ and Na_3_PO_4_, to analyze the influence of the grain volume on Na-ion transport. In the case of the oxide, high GB resistance was confirmed with the Na-ion conductivity decreasing with decreasing grain volume. In contrast, for Na_3_PS_4_, the overall influence of the GBs was significantly weaker. The primary reason for this difference was ascribed to the minimal change in the local structures and Na-ion conduction mechanism between bulk and polycrystalline Na_3_PS_4_ compared to Na_3_PO_4_, where the change is more dramatic, and there is evidence of the overcoordination of Na ions at the GBs.

In a subsequent study, Shen et al.^79^ utilized DFT and AIMD simulations to assess the relationship between GB orientation and Na vacancy concentration in the context of Na-ion diffusion in cubic Na_3_PS_4_. It was found that the blocking effects in GB cores strongly depend on the vacancy distribution in the GB structures, which in turn is determined by the Na vacancy segregation energy and the segregation sites available at the GB cores. The segregation energy of Na vacancies was defined as the difference between their formation energies in the GB and in the bulk. It was predicted that GBs with higher Na vacancy segregation energy and fewer trapping sites, i.e., locations where Na-ion transport is blocked, at their cores exhibit higher ionic diffusivity in polycrystalline Na_3_PS_4_. The importance of GB engineering, possibly by tuning their tilt angles or doping, in achieving competitive solid electrolytes was also highlighted by the authors. The interactions between point defects and GBs in Na_3_PS_4_ were again explored by Wang et al.^80^ using a combination of DFT and phase field modeling. The study confirmed the anisotropic effect of GBs on ionic diffusion in the material with a strong preference for Na-ion diffusion along the GBs (energy barrier of 0.58 eV) as opposed to across them (energy barrier of 0.11 eV).

## 4 Modeling Mechanical Properties and Electronic Structure at Grain Boundaries

Contrary to predictions from earlier theoretical studies,^81^ it is now accepted that lithium and sodium dendrites can still occur in solid-state batteries when using solid electrolytes with high density and moduli. Inevitably, this discovery has led to detailed investigations into the significance that microstructural features, including GBs, pores, and cracks, in solid electrolytes may have in dendrite nucleation. Using prominent examples from the literature, here, we discuss how state-of-the-art atomistic modeling can be used to reveal fundamental insights into the mechanical performance and electronic structure of GBs in solid electrolytes and predict their influence on dendrite formation and propagation in solid-state batteries.

**Figure 6:**
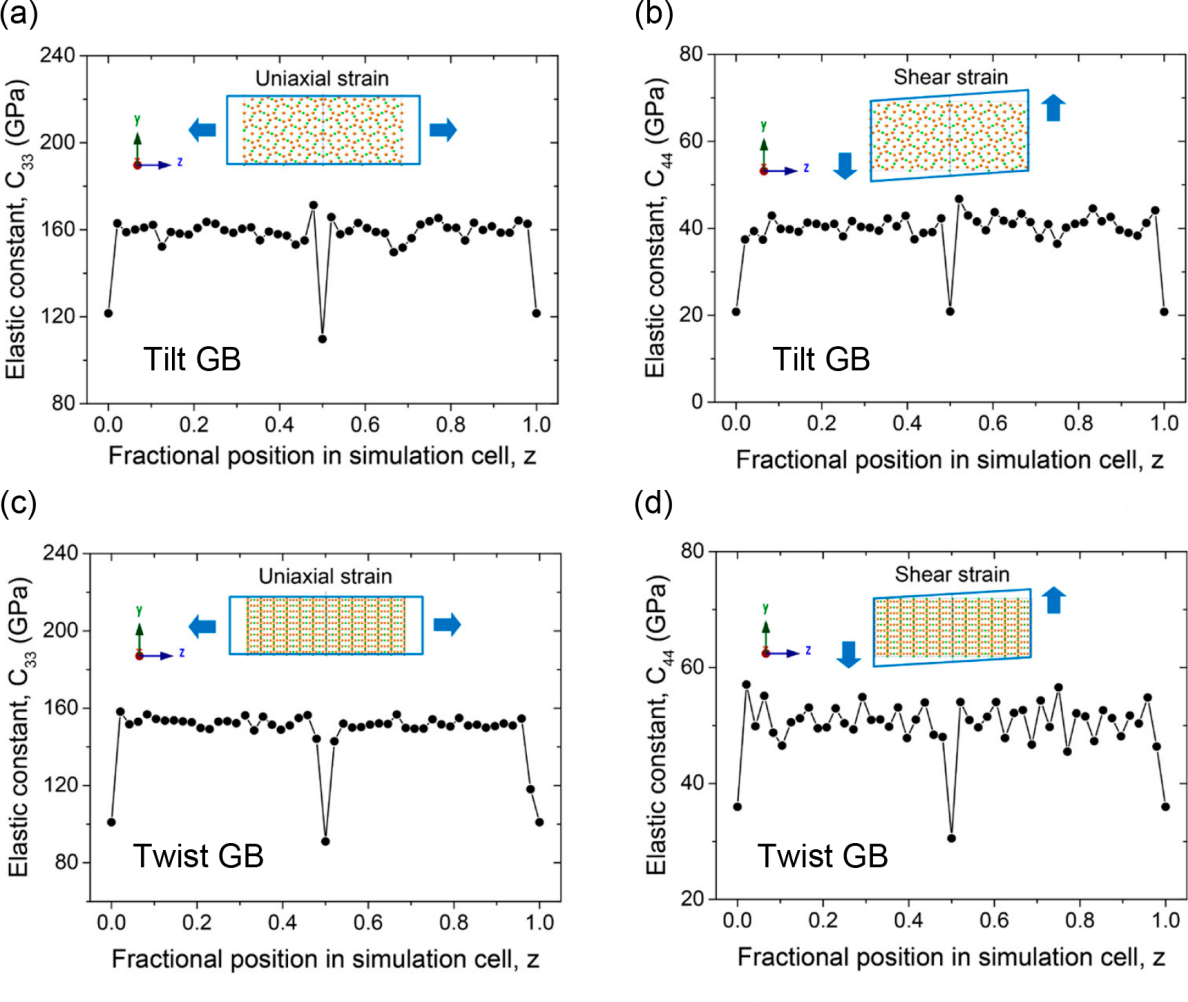
MD-calculated elastic constants *C*_33_ and *C*_44_ at 300 K as a function of position normal to the GB planes for (a, b) a Σ5 symmetric tilt GB and (c, d) a Σ5 twist GB in LLZO. Reproduced with permission from ref (82). Copyright 2018, American Chemical Society.

The first study to evaluate the mechanical properties of GBs in solid electrolytes for solid-state batteries was that of Yu and Siegel in 2018.^82^ Using LLZO as a prototype solid electrolyte, the authors carried out force field-based MD calculations on both tilt and twist GBs and reported that significant softening of its elastic properties occurs in their vicinity. As illustrated in Figure 6, two elastic constants, *C*_33_ and *C*_44_, which represent the elastic and shear moduli of the system, respectively, were calculated for the bulk and GB regions of their simulation cells. Both constants were found to be significantly reduced at the GBs, indicating that they are more susceptible to deformation and shearing than are the bulk. In fact, it was predicted that GBs in LLZO have elastic moduli that are ∼25%–50% smaller than the bulk. The softness of these GBs was identified as an important mechanism through which dendrites can penetrate ostensibly stiff solid electrolytes. The mechanical properties (Young’s, bulk, and shear moduli) of Li_3_OCl and its Σ3 and Σ5 GBs have been calculated using DFT simulations.^62^ Similar to the case of LLZO above, it was found that GBs generally increase the softness of Li_3_OCl, which is likely to impact its performance when it is in contact with electrode materials.

In the same study, Chen et al.^62^ also analyzed the electronic structures of Σ3 and Σ5 GBs in Li_3_OCl. They found that the wide bandgap of Li_3_OCl (4.78 eV) was reduced to ∼4.4 and ∼3.7 eV for the Σ3 and Σ5 GBs, respectively, suggesting that the electrochemical stability window of this polycrystalline material may be restricted when considering GB effects. Although these results were not discussed in the context of dendrites, it is expected that bandgap reductions at GBs in solid electrolytes can result in them acting as channels for leakage current.^19^ Bandgap reductions were also calculated at the GBs of Al- and Nb-doped and undoped LLZO in later computational studies.^72,73^ Furthermore, excess electrons were found to localize at the ZrO_5_ units at the GBs of LLZO, and it was proposed that under the influences of a Li metal anode and bias voltage during Li plating (charging), these excess electrons could become delocalized and show high transport behavior, potentially resulting in Li dendrite penetration.^72^ Three representative model structures for intergranular regions, i.e., a stoichiometric GB, an A-site deficient GB, and an intergranular pore, were simulated for another oxide solid electrolyte, namely, LLTO.^83^ While the stoichiometric GB region exhibited electronic insulation, the A-site deficient GB region was found to display considerable electronic conductivity. In the intergranular pore structure, Li ions preferentially enter as neutral species and present a p-type conductivity. As a result, Li dendrite nucleation was predicted to begin in the intergranular pore spaces of LLTO.

**Figure 7:**
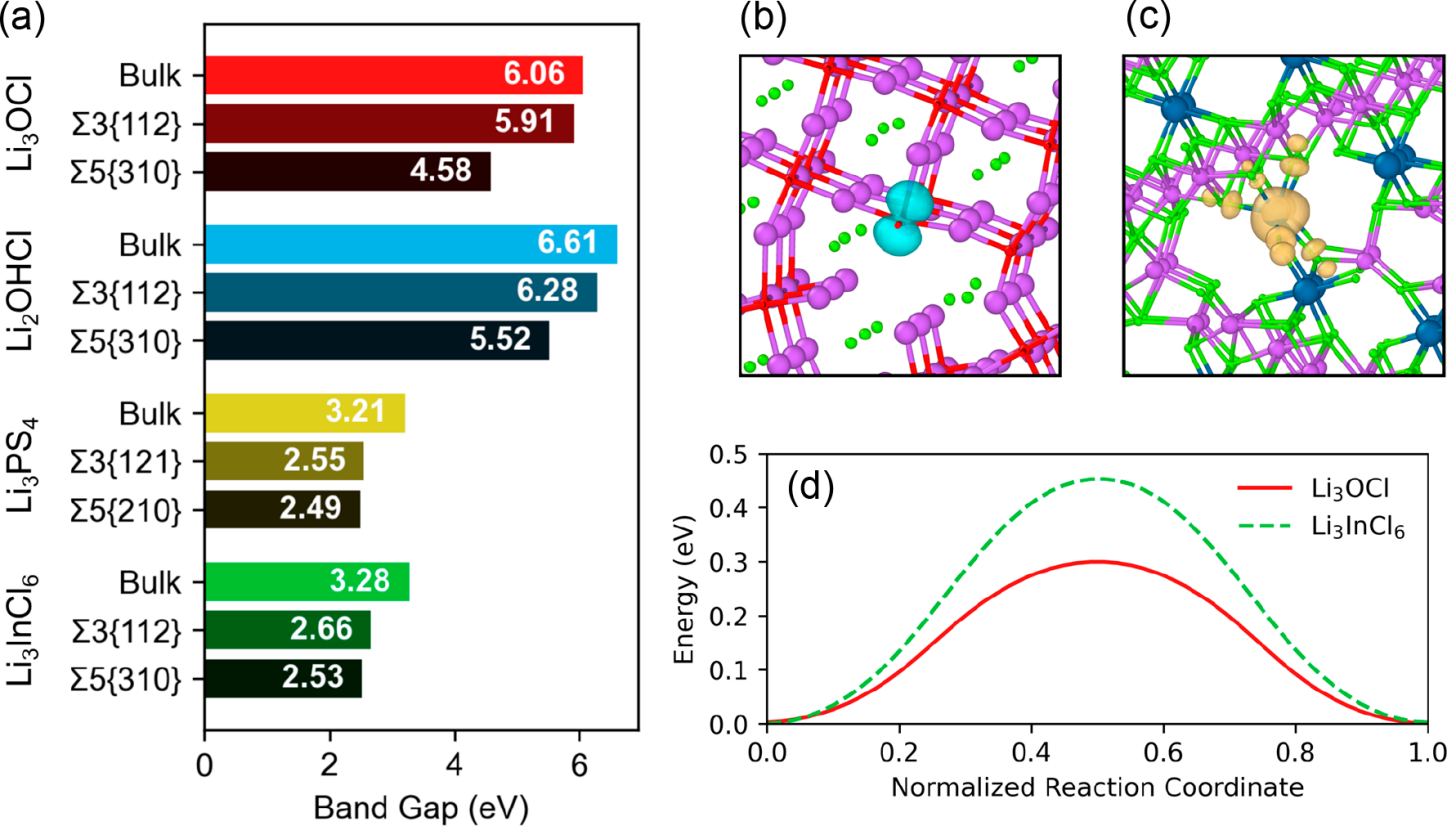
(a) Calculated bandgaps of various solid electrolytes in the bulk and in the vicinity of GBs. Isosurface plots of (b) a hole polaron in Li_3_OCl and (c) an electron polaron in Li_3_InCl_6_. (d) Adiabatic potential energy surface associated with the hopping of each polaron. Reproduced with permission under a CC BY 4.0 license from ref (18). Copyright 2023, Wiley-VCH.

In our very recent work, we determined the influence of GBs on the electronic structure and conductivity of four different solid electrolytes, namely, Li_3_OCl, Li_2_OHCl, Li_3_PS_4_, and Li_3_InCl_6_.^18^ As shown in Figure 7(a), the considered GBs exhibit reduced bandgaps to different degrees in the vicinity of the boundary. These reductions corresponded to states appearing above the valence band maximum, with the exception of the Li_3_OCl Σ5(310) and Li_3_InCl_6_ Σ3(112) GBs, where there are significant contributions from states appearing below the conduction band minimum. In contrast to previous studies, our work explicitly showed the role that polaron diffusion can play in the leakage current at GBs in solid electrolytes. Specifically, we examined the examples of a hole polaron localized at the Li_3_OCl Σ3(310) GB (Figure 7(b)) and an electron polaron in Li_3_InCl_6_ Σ3(112) GB (Figure 7(c)) and calculated their polaron hopping rates using Marcus–Emin–Holstein–Austin–Mott theory. As displayed in Figure 7(d), Li_3_OCl and Li_3_InCl_6_ GBs exhibited adiabatic activation energies of 0.30 and 0.45 eV, respectively, with corresponding hopping rates at 300 K of 4.1 × 10^8^ and 6.4 × 10^5^ Hz. These findings are discussed in detail in the context of dendrite formation and important synthesis considerations for solid electrolytes.

## 5 Conclusions and Outlook

By highlighting important recent studies in the literature, this Perspective has illustrated the power and versatility of the atomistic modeling of GBs in solid electrolytes for solid-state batteries. We have discussed how both first-principles (e.g., DFT and AIMD) and force field-based (e.g., MD) methods have been widely utilized to reveal the properties of GBs at scales not yet accessible experimentally. In particular, the contribution that GBs have in determining and changing the overall ionic conductivity and mechanisms in different types of solid electrolytes has been presented by using key examples. For example, the fundamentally different behavior of GBs resulting from the different anions (oxide, sulfide, and halide) in the electrolyte and how the reorientation dynamics of anion groups can be impacted at the GBs have been disclosed. Another pivotal area is the implementation of modeling in determining the mechanical and electronic properties of GBs and how they contribute to dendrite growth and degradation in solid-state batteries are considered. For example, it has been shown that the softness of GBs is a crucial parameter in determining whether dendrites can penetrate into a solid electrolyte and that polaron diffusion at GBs can be an important consideration in the context of leakage current.

We now address potential future trends, opportunities, and challenges for the atomistic simulation of GBs in the context of understanding and developing state-of-the-art solid electrolytes and solid-state batteries. Although the application of atomistic simulations in this field remains in its relative infancy, with all relevant studies having been published in the last six to seven years, we anticipate a rapid surge in the number of publications featuring explicit GB simulations in the near future. Furthermore, we do not believe that this growth will be limited only to solid electrolytes or indeed battery materials. Polycrystalline materials are central to energy storage, generation, and conversion technologies, and their simulation is therefore vital for enhancing material and device performance and stability. The development of more efficient and powerful MLFFs is likely to play an important role in this regard. While some studies have begun to develop MLFFs for individual GBs, as discussed above, there are currently no reports of such models for explicit polycrystals. Given the inherent disorder and range of chemical environments present in such systems, this task will inevitably be challenging but could offer an exciting route to the understanding and design of polycrystalline energy materials. We also expect machine learning approaches to be applied to interfaces, including GBs, more generally, as they have been to readily predict, discover, and understand materials at the bulk scale.

In the context of battery solid electrolytes, the pool of Li-based materials so far considered regarding GB simulations has been limited to only a selection of oxides (LLZO, Li_3_OCl, Li_2_OHCl, LLTO, LSTZ0.75, and LZP), two sulfides (Li_10_GeP_2_S_12_ and Li_3_PS_4_), and one halide (Li_3_InCl_6_). In addition, only two Na-based materials have been investigated, namely, Na_3_PO_4_ and Na_3_PS_4_. With the continuing development and increasing capability of atomistic modeling techniques and the new families of solid electrolytes being reported regularly, it is expected that the variety of materials that will be investigated in future will exponentially increase. This also applies to solid electrolytes with different mobile ions, including K^+^, Mg^2+^, Ca^2+^, and Zn^2+^, where very little known is about their GB behavior, particularly at the atomic scale. This is also likely to be the case for the range of challenges that will be tackled. For example, we expect more studies to focus on how important transport phenomena in bulk solid electrolytes, such as the paddlewheel effect, correlated ion transport, and polaron hopping, change at GBs. The atomistic modeling of large-scale microstructural features, such as pores and cracks, in solid electrolytes also represents an exciting avenue to explore. Our research group is actively working in all of these areas, and we continue to drive the development of atomistic modeling for GBs (and other interfaces) in energy materials with the expectation that it becomes prevalent within the global materials modeling community.
